# An Unusual Mimic of Axial Spondyloarthritis: A Case Report

**DOI:** 10.7759/cureus.61441

**Published:** 2024-05-31

**Authors:** Sharan Bose, Kavitha Mohanasundaram, Kandasamy Venkataraju Rajalakshmi, Ananthakumar Perumal Kumaresan, Jibin Simon

**Affiliations:** 1 Internal Medicine, Saveetha Institute of Medical and Technical Sciences, Saveetha University, Chennai, IND; 2 Rheumatology, Saveetha Institute of Medical and Technical Sciences, Saveetha University, Chennai, IND

**Keywords:** sacroiliitis-like presentation, axial spondyloarthritis, inflammatory back pain, vitamin-d deficiency, normocalcemic hyperparathyroidism

## Abstract

Axial spondyloarthritis (SpA) is a chronic inflammatory condition predominantly affecting the sacroiliac joints and spine, typically presenting before the age of 45 years with inflammatory back pain. However, diagnostic challenges arise when atypical features and negative autoimmune markers obscure the clinical picture. We present a case of a male in his 40s with no significant medical history, presenting with a three-month history of inflammatory back pain. Despite negative human leukocyte antigen B27 (HLA-B27) status, clinical examination, including positive findings on the FABER (flexion, abduction, and external rotation) test and exaggerated muscle tenderness, raised suspicion of axial SpA. An MRI of the pelvis confirmed bilateral symmetrical sacroiliitis, supporting the diagnosis. Unexpectedly, further investigations revealed a very low vitamin D level, normal calcium levels, and elevated parathyroid hormone (PTH), suggesting secondary hyperparathyroidism. A subsequent PET scan disclosed increased uptake posterior to the right lobe of the thyroid, prompting consideration of secondary hyperparathyroidism due to severe vitamin D deficiency. Treatment with vitamin D supplementation and nonsteroidal anti-inflammatory drugs yielded remarkable improvement in symptoms, with normal repeat blood investigations post-treatment. This case underscores the importance of a comprehensive diagnostic approach in patients with inflammatory back pain, especially when classical markers such as HLA-B27 are negative. It highlights the potential interplay between axial SpA and secondary hyperparathyroidism, emphasizing the need for vigilance and interdisciplinary collaboration in clinical practice.

## Introduction

Axial spondyloarthritis (SpA) is a chronic inflammatory condition primarily affecting the sacroiliac joints and the spine. It typically manifests with insidious onset back pain, often striking individuals before the age of 45 years. The hallmark of this pain is its improvement with physical activity like walking and exercise, but little to no relief with rest [1]. The diagnostic journey of a patient with axial SpA often heavily relies on magnetic resonance imaging (MRI), particularly for detecting sacroiliitis coupled with adjacent bone marrow edema, which is considered classical for active axial SpA. However, the challenge arises from the fact that numerous other conditions can produce similar clinical presentations and MRI findings. Conditions such as Paget’s disease, sarcoidosis, hyperparathyroidism, brucellosis, and even diffuse idiopathic skeletal hyperostosis (DISH) in elderly diabetic individuals can mimic the symptoms and radiological features of axial SpA.

This diagnostic conundrum underscores the importance of a comprehensive approach, wherein clinicians must integrate clinical assessment, imaging findings, and laboratory investigations to arrive at an accurate diagnosis. While the presence of multiple spondyloarthritis clinical features is suggestive of axial SpA, it is not sufficient for a definitive diagnosis. Differential diagnosis remains crucial, especially when faced with ambiguous clinical presentations or overlapping symptoms.

In this context, we delve into a specific case report to illustrate the challenges encountered in diagnosing inflammatory back pain, exploring the intricacies of differential diagnosis and the critical role of MRI imaging in distinguishing axial SpA from its mimics. Through this exploration, we aim to shed light on the complexities of diagnosing axial SpA and the importance of considering a broad differential to ensure optimal patient care and management.

## Case presentation

A 44-year-old male with no co-morbidities presented with a history of low backache of three months duration, more in the mornings, and with no sensory or motor deficits. The pain subsided after around one hour of activity in the morning. He had no history of trauma or fever. No past clinical history of psoriasis, tuberculosis, other joint pains, or inflammatory bowel disease. There was no significant family history of similar complaints or other connective tissue disorders. On examination, he was conscious, oriented, and afebrile. His vitals were stable. Neurological examination revealed no focal neurological deficits. Local examination did not elicit tenderness over the vertebrae or sacroiliac joints, but the FABER (flexion, abduction, and external rotation) test was positive, indicating potential involvement of the hip joint. Additionally, muscle tenderness was noted during the FABER test, further suggesting inflammatory involvement. The straight leg raising test was negative.

**Figure 1:**
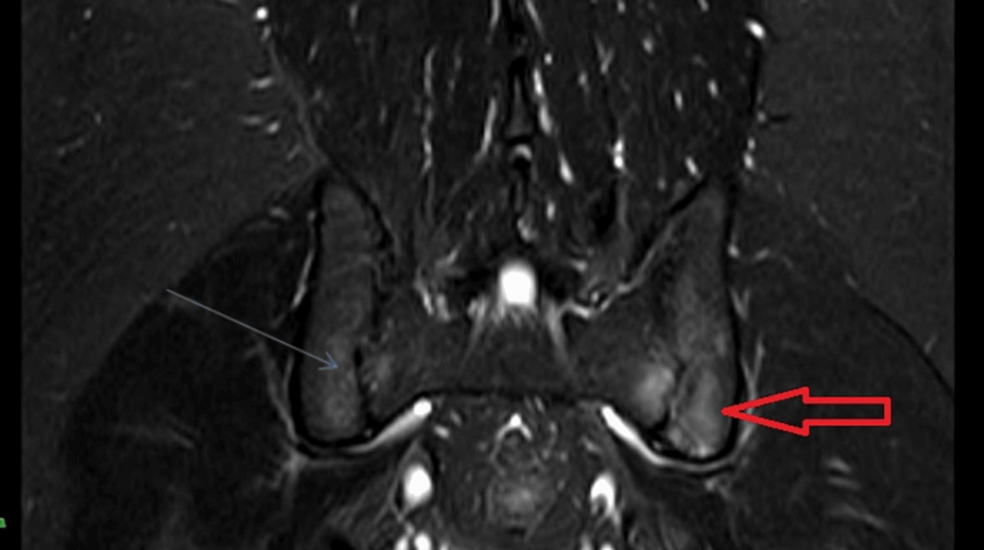
MRI (STIR image) of the pelvis showed bilateral sacroiliitis (left > right). STIR: short tau inversion recovery.

An MRI of the pelvis (Figure 1) was performed, revealing features suggestive of active sacroiliitis with erosions, supporting the diagnosis of axial SpA with an Assessment of Spondyloarthritis International Society (ASAS) criteria score of 2. Human leukocyte antigen B27 (HLA-B27) was, however, negative. Despite the negative HLA-B27 status, the clinical presentation and imaging findings were highly indicative of this condition. However, additional investigations revealed unexpected findings. Blood tests showed very low vitamin D levels and raised parathyroid hormone (PTH) levels, raising suspicion of secondary hyperparathyroidism (Table 1).

**Table 1:** A summary of relevant laboratory investigations done.

Parameter	On initial workup	After treatment	Reference range
Calcium	8.80 mg/dl	9.20 mg/dl	8.4-10.2 mg/dl
Vitamin D	9.04 ng/ml	43 ng/ml	Deficiency: <10 ng/ml
Parathyroid hormone	474.4 pg/ml	128.6 pg/ml	15-88 pg/ml
Phosphorus	2.60 mg/dl	3.20 mg/dl	2.5-4.5 mg/dl
Serum albumin	4.1 g/dl	4.0 g/dl	3.5-5 g/dl
Urea	28 mg/dl	24 mg/dl	15-40 mg/dl
Creatinine	0.8 mg/dl	0.8 mg/dl	0.6-1.0 mg/dl

A high-resolution computed tomography (HRCT) of the chest was also done (as part of a treatment work-up for axial spondyloarthritis), and it showed lytic areas in the manubrium sternum with a healed fracture of the left 6th rib. In view of the lytic lesions, suspected Brown’s tumor, and a raised PTH, a diagnosis of hyperparathyroidism was made. However, normal calcium and a very low vitamin D prompted us to look for evidence of malignancy too. The PET scan revealed increased uptake posterior to the right lobe of the thyroid gland, consistent with a parathyroid adenoma, along with other findings corroborating the diagnosis of hyperparathyroidism and Brown’s tumor. The identification of these lesions prompted collaboration with an endocrinologist for further evaluation and management.

The patient’s management plan after a consultation with the endocrinologist involved the initiation of vitamin D replacement therapy to address the deficiency. Remarkably, significant symptomatic relief was reported following the initiation of treatment. Subsequent follow-up (after six weeks) revealed a reduction in PTH levels (128.6 pg/ml), indicating a positive response to therapy.

## Discussion

Axial SpA encompasses a spectrum of chronic inflammatory conditions primarily affecting the axial skeleton, including the spine and sacroiliac joints. Patients typically present before the age of 45 years with inflammatory back pain characterized by nocturnal and morning stiffness. This pain improves with activity but persists with rest. The ASAS classification criteria for SpA requires either sacroiliitis on imaging and one or more SpA features (see below) OR HLA-B27 positivity and two or more SpA features. SpA features include inflammatory back pain, enthesitis, arthritis, uveitis, dactylitis, a good response to nonsteroidal anti-inflammatory drugs, a positive family history of SpA, HLA-B27, and an elevated C-reactive protein (CRP). Genetic predisposition, particularly the presence of the HLA-B27 allele, is strongly associated with axial SpA. Some forms are associated with psoriasis and inflammatory bowel disease (IBD).

The advent of MRI of the sacroiliac joints has revolutionized the diagnosis of axial SpA, allowing for early detection through the identification of bone marrow edema [2]. Additional radiological evidence of sacroiliitis includes blurring of joint margins, erosions, sclerosis, and narrowing of the joint space [3]. Despite these advancements, the diagnosis of axial SpA remains challenging, as several conditions can mimic its clinical presentation. The various causes of unilateral sacroiliitis include infections (tuberculosis, brucellosis, or pyogenic), the SAPHO (synovitis, acne, pustulosis, hyperostosis, and osteitis) syndrome, neoplasms, etc. Causes for bilateral sacroiliitis are axial SpA, rheumatoid arthritis, osteitis condensans ilii, enteropathic arthritis, and notably, hyperparathyroidism [4].

Hyperparathyroidism presents a unique challenge as a mimic of axial SpA. It may occur due to a disorder within the parathyroid glands (primary) or as a response to external stimuli (secondary) or rarely an autonomous adenoma (tertiary) [5]. Common signs and symptoms include abdominal pain, constipation, kidney stones, lethargy, depression, and bone pain. This excess PTH stimulates osteoblasts and osteoclasts, resulting in bone resorption [6] and the release of calcium from the bone. It also acts on the kidneys via vitamin D to increase calcium absorption by the nephron. Diagnosis of hyperparathyroidism relies on PTH immunoassay, serum calcium, phosphorous, and vitamin D levels. Management of hyperparathyroidism focuses on addressing the underlying cause, which may involve surgical intervention for primary or tertiary hyperparathyroidism, along with medical management of associated complications including hypercalcemia.

Distinguishing hyperparathyroidism from axial SpA requires careful consideration of clinical and laboratory findings. The absence of HLA-B27 positivity, preserved bone mineral density, and relative sparing of the sacroiliac joint can provide useful clues. However, in cases where predominant back pain and sacroiliac joint involvement confound the diagnosis, a comprehensive approach is essential for accurate differentiation and appropriate management.

## Conclusions

In summary, while axial SpA remains a leading cause of inflammatory back pain, clinicians must remain vigilant for mimics such as hyperparathyroidism. A thorough understanding of the clinical characteristics, diagnostic approach, and management strategies for both conditions is paramount in providing comprehensive care to patients presenting with axial skeletal involvement and inflammatory symptoms. The fact that the treatment for hyperparathyroidism is so different (and often curative) from that of other inflammatory causes means that appropriate due diligence must be done in ruling it out.
